# Immune Dysregulation in Endometriomas: Implications for Inflammation

**DOI:** 10.3390/ijms25094802

**Published:** 2024-04-28

**Authors:** Izabela Dymanowska-Dyjak, Barbara Terpiłowska, Izabela Morawska-Michalska, Adam Michalski, Grzegorz Polak, Michał Terpiłowski, Mansur Rahnama-Hezavah, Ewelina Grywalska

**Affiliations:** 1 Independent Laboratory of Minimally Invasive Gynecology and Gynecological Endocrinology, Medical University of Lublin, 20-081 Lublin, Poland; i.dymanowska@gmail.com (I.D.-D.); polakg@yahoo.com (G.P.); 2 Department of Gynecological Oncology and Gynecology, Medical University of Lublin, 20-081 Lublin, Poland; barb.terpilowska@gmail.com; 3 Department of Clinical Immunology, Medical University of Lublin, 20-093 Lublin, Poland; izabelamorawska19@gmail.com (I.M.-M.); michalski.g.adam@gmail.com (A.M.); 4 Department of Vascular Surgery and Angiology, Medical University of Lublin, 20-081 Lublin, Poland; michal.terpilowski@gmail.com; 5 Chair and Department of Oral Surgery, Medical University of Lublin, 20-093 Lublin, Poland; mansur.rahnama-hezavah@umlub.pl; 6 Department of Experimental Immunology, Medical University of Lublin, 20-093 Lublin, Poland

**Keywords:** endometriosis, endometrioma, endometrioidal cyst, chocolate cyst, immune dysregulation, inflammation

## Abstract

The most common manifestation of endometriosis, a condition characterized by the presence of endometrial-like tissue outside of the uterus, is the endometrioma, a cystic ovarian lesion. It is a commonly occurring condition associated with chronic pelvic pain exacerbated prior to and during menstruation, as well as infertility. The exact pathomechanisms of the endometrioma are still not fully understood. Emerging evidence suggests a pivotal role of immune dysregulation in the pathogenesis of endometriomas, primarily influencing both local and systemic inflammatory processes. Among the factors implicated in the creation of the inflammatory milieu associated with endometriomas, alterations in both serum and local levels of several cytokines stand out, including IL-6, IL-8, and IL-1β, along with abnormalities in the innate immune system. While numerous signaling pathways have been suggested to play a role in the inflammatory process linked to endometriomas, only NF-κB has been conclusively demonstrated to be involved. Additionally, increased oxidative stress, both resulting from and contributing to endometriomas, has been identified as a primary driver of both systemic and local inflammation associated with the condition. This article reviews the current understanding of immune dysfunctions in the endometrioma and their implications for inflammation.

## 1. Introduction

Endometriosis is defined as a chronic condition characterized by the presence of endometrial glands and stroma outside of the uterine cavity [1]. Classified as an estrogen-dependent inflammatory condition, the severity of the symptoms, most notably dyspareunia and dysmenorrhea, exacerbate days prior to and during menstrual bleeding [2]. Endometriosis is one of the most common gynecological conditions, affecting approximately 5–10% of women of reproductive age [3]. Furthermore, it is suspected that the condition is significantly underdiagnosed and the real prevalence rate might be significantly higher [4]. Indeed, due to its prevalence, endometriosis not only is the primary cause of pelvic pain in women but also one of the major causes of decreased fertility. Endometriosis can be divided into three primary subtypes: peritoneal endometriosis, ovarian endometriosis, and deep invasive endometriosis. Out of the three, ovarian endometrial cysts, often called endometriomas or chocolate cysts, due to the presence of dark red-brown endometrial fluid within them, are the most common presentation, accounting for approximately 17–44% of all endometriosis cases [5]. Despite the prevalence, treatment options are, however, severely limited, as pharmacological treatment is largely ineffective or causes significant adverse effects, thus relying primarily on surgical treatment and, in the case of infertility associated with endometriosis, in vitro fertilization (IVF) [6].

Unfortunately, the pathogenesis of endometriosis is still not fully understood. Several hypotheses aiming to explain this process have been formed, but none of them has, however, fully explained this phenomenon. The retrograde menstruation theory, also known as Sampson’s theory, suggests that, during menstruation, some endometrial tissue travels retrogradely through the fallopian tubes, where it implants itself in the surrounding tissues, leading to endometrial growth [7]. While widely accepted, this theory primarily accounts for the presence of peritoneal and ovarian endometriosis (OE); it does not fully explain the existence of deep invasive endometriosis [8]. Moreover, it does not account for cases of endometriosis with onset prior to the beginning of menstruation, as well as in women with Mayer-Rokitansky-Küster-Hauser syndrome [1]. Additionally, it fails to explain the extremely rare occurrences of male endometriosis [9]. The coelomic metaplasia theory, also known as Gruenwald’s theory, implies that that peritoneal serosa and serosa-like structures, being embryologically related to Mullerian ducts, have the potential to undergo metaplastic transformation into endometrial-like tissue, leading to the development of endometriosis [10]. This theory can explain the cases mentioned above, for which the retrograde menstruation theory cannot be applied. The embryogenetic theory suggests that endometrial tissue, in response to the exposure to estrogen, may develop from embryonic remnants that retain the ability to differentiate into endometrial-like tissue, thus forming endometriotic lesions [11]. A similar theory is the stem cell hypothesis, which suggests that both endometrial and hematopoietic stem cells retain the ability to differentiate into endometrial cells [12]. This process could occur in various anatomical sites, potentially leading to the formation of endometriotic lesions. The hematogenous and/or lymphatic spread theory suggests that endometriosis may share similarities with cancer, as it implies the potential for endometrial cells to disseminate through the bloodstream and/or lymphatic vessels [13]. However, the paucity of substantial supporting data currently renders this theory unlikely (Table 1) [1].

Another theory and the focus of this article is the immune dysregulation theory, also known as the inflammatory theory [14]. It suggests that dysfunction of the apoptotic mechanisms responsible, among other things, for the elimination of improperly located cells partially contributes to the development of endometriosis. Malfunctioning of these mechanisms leads to a lack of response to the appearance of endometrial cells, followed by the stimulated development of endometrial lesions by cells such as macrophages or natural killer (NK) lymphocytes [8,13,14]. The full spectrum of immune dysregulation present in patients with endometriomas is, however, much broader. In this article, we describe the current evidence of immunological dysfunctions involved in establishing the inflammatory milieu associated with the presence of endometrial cysts.

## 2. Cytokines and Chemokines

### 2.1. Interleukin-6

Analysis of blood serum in patients with endometrial cysts revealed significantly elevated levels of interleukin-6 (IL-6) in both blood serum as well as in the endometrioma and its surrounding tissues [15,16,17,18,19,20]. IL-6 is one of the cytokines implicated in both the establishment and progression of the disease, exerting its action via a number of mechanisms. Notably, IL-6 impairs the cytotoxic function of NK cells through the modulation of Src homology region 2 domain-containing phosphatase-2 (SHP-2), consequently decreasing their reactivity toward endometriotic cells [21]. Additionally, IL-6 induces the shedding of intercellular adhesion molecule-1 (ICAM-1) from endometriotic cells [22]. As ICAM-1 plays a crucial role in NK cell-mediated cytotoxicity, this shedding further diminishes the reactivity of NK cells toward endometriotic cells [22,23]. Importantly, IL-6 mediates numerous inflammatory signals of both innate and adaptive systems, taking part in establishing the systemic and local inflammation associated with endometrial cysts [24]. IL-6, together with soluble IL-6 receptor α (sIL-6Rα), is also essential for transitioning established acute inflammation into a chronic state by promoting the shift from a neutrophilic leukocyte infiltrate, typical of acute inflammation, to a monocyte/macrophage-dominated one, characteristic of chronic inflammation [25]. Lastly, elevated serum IL-6 levels are linked to decreased fertility and higher rates of unsuccessful pregnancies, potentially through suppression of blastocyst formation, as observed in mouse models [26,27,28]. Researchers showed that laparoscopic removal of the ovarian endometrium reduced serum IL-6 concentrations to baseline levels, suggesting that the increased serum IL-6 concentrations may originate from the ovarian endometrioma [16].

### 2.2. Interleukin-8

Similarly to IL-6, elevated levels of interleukin-8 (IL-8) have been discovered in blood serum as well as in endometriomas and surrounding tissues [17,18,29]. IL-8 is a proinflammatory cytokine, thus taking part in establishing the previously mentioned inflammatory milieu [30]. Moreover, IL-8 is known to strongly recruit neutrophils that, subsequently, produce significant amounts of interleukin-17A (IL-17A) and vascular endothelial growth factor (VEGF) [31,32]. VEGF expression is positively correlated with endometrioma size and bilateralism of cysts [33]. Scientists have found that IL-8 from peritoneal fluid, levels of which are elevated in women with endometriosis, enhances proliferation of stromal cells derived from ovarian cysts [34]. It seems that IL-8 works together with tumor necrosis factor α (TNF-α) in a mechanism through which TNF-α stimulates IL-8 gene and protein expression through NF-κB activation in endometriotic stromal cells [34,35]. This abnormality can be corrected with gonadotropin-releasing hormone agonist (GnRHa) treatment, enhancing the expression of IL-8 indirectly by reducing TNF-α-induced NF-κB activation [35]. The probable involvement of IL-8 in the etiopathogenesis of cysts in endometriosis may be supported by the fact that scientists have discovered that women with higher concentrations of IL-6 and IL-8 in the fluid collected from cysts have earlier recurrence of endometrioma symptoms [36]. An interesting observation is that levels of IL-8 and monocyte chemoattractant protein-1 (MCP-1) remained higher in fluid collected from an ovary affected by endometriosis compared to that in women without the disease. The second ovary, unaffected by endometriosis, also had average levels of this cytokine [20]. Women with ovarian endometriomas tend to have higher levels of IL-6 and IL-8 in serum as well, and the serum level of IL-8 has high predictive value for the presence of OE. The authors of the paper suggest that the levels of these cytokines may help to distinguish the occurrence of exclusively ovarian endometriomas from deep infiltrating endometriosis [18]. Interestingly, another scientific group described no differences in IL-8 mRNA expression in ovarian endometriotic tissue compared to the control group [37].

### 2.3. Interleukin-1β

Interleukin-1β (IL-1β), also known as lymphocyte activating factor, is a pro-inflammatory interleukin believed to play a potential role in ovarian endometriomas. In vitro studies indicate that IL-1β, along with TNF-α, can indirectly stimulate the expression of IL-6 and protease-activated receptor-2 (PAR-2) mRNA through activin A, which increases the proliferation of endometrial stromal cells [38]. This cytokine enhances the expression of tryptophan 2,3-dioxygenase (TDO), which possesses immune tolerance-silencing properties. Consequently, it stimulates tryptophan catabolism while also triggering the production of IL-6 and IL-8 in endometrial stromal cells [39]. Levels of IL-1β were found to be elevated in fluid collected from endometrioma-affected ovaries compared to that in ovarian fluid from women without endometriosis. Notably, even the second unaffected ovary from endometriosis patients exhibited mild levels of IL-1β [20]. In an in vitro model, IL-1β was observed to stimulate the expression of thymic stromal lymphopoietin (TSLP) mRNA and the secretion of this protein from cells in primary cultures of endometrial stromal cells. TSLP is a protein that influences the immune response by inducing the polarization of cells toward the Th2 response. This observed mechanism was influenced by external factors in the study—for example, interleukin-4 (IL-4) increased the secretion of TSLP induced by interleukin-1 (IL-1), while interferon gamma (IFN-γ) reduced it. TSLP levels were higher in plasma and peritoneal fluid in women with endometriosis compared to healthy women [40]. Researchers have shown that interleukin-1β increases P21-activated inase 1 (Pak1) expression in endometrial stromal cells (ESCs) and that Pak1 immunoreactivity is increased in ovarian cysts in endometriosis. Pak1 is a protein, a kinase from the Pak family, which is postulated to be involved in the development of endometriosis. Under normal conditions, its level decreases during the secretory phase, but in women with endometriosis, this mechanism may be disturbed, leading to excessive expression of Pak1 in the eutopic endometrium [41]. Other reports of endometrial stromal cells regarding IL-1β and TNF-α indicate that these cytokines can induce activin A and follistatin mRNA and protein in culture. In this study, the concentrations of activin and follistatin and activin activity in fluid collected from the diseased ovary were also measured. The authors demonstrated the presence of follistatin and high activin A activity in endometrioma fluid, which was associated as a factor exacerbating the disease [42].

### 2.4. Other Cytokines and Chemokines

The discovered decline in levels of anti-inflammatory cytokines interleukin-19 (IL-19) and interleukin-22 (IL-22) has been suggested to help the ectopic endometrium escape from immunosurveillance [43]. Both IL-19 and IL-22 are immunosuppressive, anti-inflammatory cytokines; however, while IL-19 exerts its action by promoting Th2 cells and increasing the expression of interleukin-10 (IL-10), IL-22 primarily promotes proliferation and tissue regeneration of non-hematopoietic epithelial and stromal cells [44,45,46]. It has been therefore suggested that decreased serum levels of IL-19 and IL-22 might contribute to the endometrioma evading immunosurveillance, although they could also simply reflect an increase in levels of proinflammatory cytokines [43].

TNF-α is a pleiotropic cytokine, exerting its numerous proinflammatory effects primarily through activation of the NF-κB and MAPK pathways [47]. It has been found to be a key player not only in the induction and propagation of endometrioma-related inflammation but also for the survival of endometriotic cells as well. Through the activation of NF-κB and extracellular signal-regulated kinase 1/2 (ERK1/2), TNF-α induces abundant expression of IL-6 in endometriotic stromal cells [48,49]. Similarly, TNF-α has been found to increase the expression of IL-8 mRNA within cultured endometrial cells in vitro, as well as IL-8 protein. Moreover, the levels of IL-8 protein correlated with increased proliferation of endometriotic cells, suggesting that TNF-α stimulates proliferation indirectly via the aforementioned interleukin [34]. Investigation of the cyst microenvironment established elevated levels of TNF-α in endometrial flushing fluid in women diagnosed with ovarian endometriomas, further indicating the inflammation of surrounding tissues [50].

IL-4 is another cytokine potentially involved in the establishment and development of endometrial cysts. One study revealed that immunohistochemical staining in patients who underwent surgical removal of endometriomas showed numerous IL-4 (+) cells in the stroma. The authors suggested that locally produced IL-4 may contribute to the disease’s development. The authors of this study demonstrated that IL-4 stimulated the phosphorylation of numerous MAPK proteins, and the addition of inhibitors for these MAPKs suppressed IL-4-induced ESC proliferation. An intriguing observation is the synergistic action of interleukin 4 with TNF-α [51]. In another study, ovarian biopsy with fluid collection was performed during surgery in women undergoing ovarian endometrioma surgery or sterilization without disease. The authors then looked for the presence of mRNA for individual cytokines. It was shown that IL-6, IL-10, and IL-1α mRNA were present in samples from most patients, but not healthy women. Interestingly, the expression of IL-8, interleukin-13 (IL-13), IFN-γ, and TNF-α mRNA was not statistically different, while interleukin-2 (IL-2) and IL-4 mRNA were not expressed in any of the groups [37].

The role of proinflammatory interleukin-16 (IL-16) is still yet to be fully explained. The studies using a mice model of endometriosis have suggested that the iron overload associated with the environment of endometriomas triggers gasdermin-E-mediated pyroptosis and the release of active IL-16. It is of the most importance, as the authors discovered that IL-16-knockout mice tended to have less pronounced inflammation and decreased endometriotic lesion weight. Moreover, IL-16 levels seemed to correlate with IL-1β and IL-6 cystic fluid levels. The authors postulated that, as such, IL-16 might play a pivotal role in initiating endometrioma-related inflammation [52].

Another cytokine with a postulated role in the immunology of endometrial cysts is transforming growth factor β1 (TGF-β1), the average serum levels of which are higher among women with endometriomas compared to those with peritoneal involvement, and they differ depending on the stage of the disease. Moreover, the plasma levels of TGF-β1 were higher in patients with endometriomas than in other types of endometriosis or the control group, thus making the authors postulate that they can be considered as an additional diagnostic marker of endometriomas [19].

Evaluation of cystic fluid revealed increased VEGF levels within endometrial cyst fluid, which not only further confirmed the observed local inflammation but also pointed to the role of the increased angiogenesis of endometriotic tissues potentially contributing to their growth and development [31]. Moreover, evaluation of biopsies of unaffected peritoneum taken from patients diagnosed with endometriomas revealed increased VEGF immunoreactivity compared to the control group, with a positive correlation observed between the size of the endometrioma and intensity of VEGF labeling [53]. As suggested, it might be related to an increase in VEGF production by activated endometrioma-associated macrophages [54]. Due to VEGF being a proinflammatory factor responsible for increasing endothelial permeability, increased VEGF immunoreactivity in the peritoneum might be associated with concurrent inflammation of the peritoneum, even when it is not affected by endometriosis [55]. Kajdos et al. highlighted elevated levels of microvesicles containing VEGF in the blood serum of patients diagnosed with endometriomas. This finding aligned with the majority of reports suggesting systemic inflammation concurrently occurring with endometriomas [56]. Of particular interest, Tan et al. presented findings that partially contradicted the previously described data. Their report indicated that VEGF levels within endometrial tissue are even lower than those observed in the eutopic endometrium [57].

**Figure 1:**
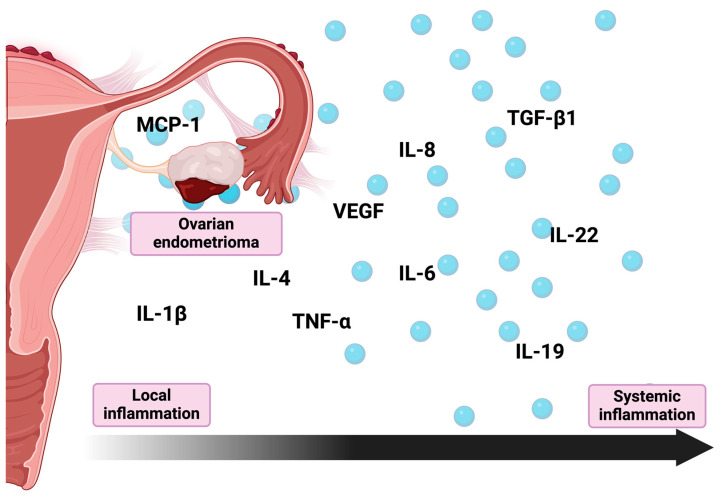
Cytokines and chemokines involved in inflammatory process associated with endometriomas. IL-1β—Interleukin 1β; IL-4—interleukin 4; IL-6—interleukin 6; IL-8—interleukin 8; IL-19—interleukin 19; IL-22—interleukin 22; MCP-1—monocyte chemoattractant protein-1; TNF-α—tumor necrosis factor α; TGF-β1—transforming growth factor β1; VEGF—vascular endothelial growth factor.

Lastly, elevated levels of MCP-1 in the follicular fluid taken from endometrioma-affected ovaries further indicate the existence of local inflammation [20]. MCP-1 is a potent pro-inflammatory chemokine and one of the key regulators of monocyte/macrophage migration and infiltration [58]. As such, it directly influences the establishment of the inflammatory environment of the endometrioma and can potentially promote the shift toward a monocyte/macrophage-dominated leukocyte infiltrate characteristic of chronic inflammation [59]. Moreover, as MCP-1 promotes cell adhesion and cell proliferation, it can potentially promote both the development and survival of endometriotic cells [60,61] (Figure 1).

## 3. Oxidative Stress

Oxidative stress is a significant factor responsible for the persistent inflammation present in endometrial cysts and surrounding tissues. Specifically, reactive oxygen species (ROS) are found to be notably abundantly produced in vitro in the endometriotic cyst fluid of human immortalized epithelial cells derived from ovarian endometriomas [62]. Consistently, an in vivo study of follicular fluid revealed increased levels of several oxidative stress markers, such as prostaglandin reductase 2 and Annexin A1 [63]. The role of oxidative stress in exacerbating the course of the disease was further confirmed by studies of the use of N-acetyl-L-cysteine, a strong antioxidant, as a potential therapy. Both Pittaluga et al. in a murine model and Porpora et al. in clinical evaluations observed a reduction in the size of the endometrioma when treated with N-acetyl-L-cysteine [64,65]. In contrast to the previous observations, Nakagawa et al. observed that the oxidative stress and antioxidant potential in the follicular fluid of patients with unilateral endometriomas resembled those without endometriomas [66]. The reason for this discrepancy is, however, still not explained.

**Figure 2:**
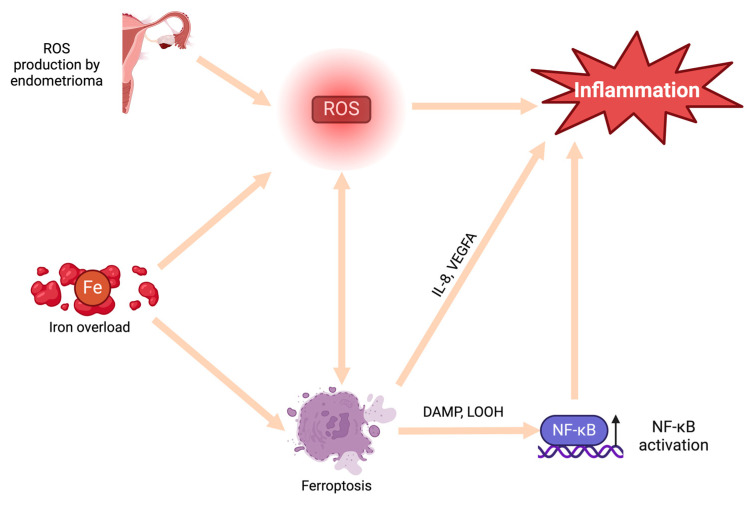
The role of oxidative stress and ferroptosis in establishing the inflammation associated with endometriomas. DAMP—damage associated molecular patterns; Fe—iron; IL-8—interleukin 8; LOOH—lipid hydroperoxides; NF-κB—nuclear factor κB; ROS—reactive oxygen species; VEGFA—vascular endothelial growth factor A.

Accumulation of menstruation-like blood within the endometrioma creates a highly proinflammatory environment, primarily attributed to the presence of iron [67,68]. Iron is known to catalyze the generation of ROS, thereby inducing oxidative stress within the endometrioma and surrounding tissues [69]. However, the proinflammatory effects of iron are not limited to only generation of ROS. Iron overload has been linked to ferroptosis, an intracellular iron-dependent form of cell death [67,70,71]. This process involves the release of damage-associated molecular patterns (DAMPs) and lipid peroxidation products, which in turn activate the NF-κB pathway, leading to inflammation [72]. Furthermore, iron overload has been correlated with increased levels of vascular endothelial growth factor A (VEGFA) and IL-8, proinflammatory cytokines described above, thus triggering inflammation through an additional mechanism [70,73,74]. However, the age of lesions directly correlates with the level of iron accumulation and, consequently, the severity of iron-associated complications [68]. There is suspicion that, over time, iron overload escalates, exacerbating the severity of associated issues. Finally, examination of cyst fluid has revealed an association between iron levels and infertility related to endometriosis [75]. Therefore, cyst fluid iron levels could serve not only as markers of inflammation but also as predictors of infertility in women with ovarian endometriomas [67,75] (Figure 2).

## 4. Immune Cells

A research group conducted an evaluation of surface markers, including RAGE, TLR-4 (recognizing HMGB1), the co-stimulation marker CD86, the activation marker CD69, and the degranulation marker CD107a, on various cells from tissue samples obtained from patients with ovarian endometriomas and those with other benign ovarian tumors. The expression of the mentioned molecules did not differ significantly on dendritic cells (DCs), macrophages, NK cells, or invariant natural killer T (iNKT) cells between the two groups. However, the expression of CD69 on both T helper (Th) and cytotoxic (Tc) lymphocytes, as well as CD107a on Tc lymphocytes, was found to be elevated in women with endometriomas. Moreover, the researchers observed that CD8+ T and CD4+ T cells were upregulated as the levels of HMGB1 (one of the damage-associated molecular patterns, or DAMPs) increased in women with endometriomas. This led the authors to suggest that the role of the adaptive immune system might be more pronounced in endometrial cysts compared to the innate immune response. In a detailed assessment, the authors described a higher percentage of M2-polarized macrophages (the M2 subtype promotes the Th2 response dependent immunosuppressive effects) and regulatory T lymphocytes in the group of women with endometrial cysts. Both of these parameters increased with HMGB1 upregulation [76]. In a mouse model of endometrioma, the authors reported that ectopic endometrial CD4+ T cells demonstrated an enhanced capacity to produce proinflammatory cytokines and exhibited upregulated proapoptotic and proinflammatory signaling pathways, such as IL-17 and TNF. Moreover, these cells seemed to be the primary source of the previously mentioned IL-16 in ovarian endometriosis, thus suggesting their pivotal role in the pathogenesis of the endometrioma [52].

In later studies, women with ovarian endometrial cysts were additionally divided into those who received preoperative dienogest therapy and those who did not. This study showed increased levels of HMGB1 and interleukin-33 (IL-33) in the peritoneal fluid of sick patients; the level of these alarmins had no significance for the advancement of the disease. In patients receiving preoperative treatment, an inverse correlation was found between the percentage of macrophages in the peritoneal fluid and the size of the tumor and r-ASRM value; both M1 and M2 fractions as well as their expression of TLR4, RAGE, and CD86 decreased along with aggravating indicators. No similar changes were observed in other cells. The authors suggested that this indicates weaker immunostimulatory activity of macrophages as EOC worsens. In the breeding experiment, the expression of TLR4, RAGE, and CD86 did not depend on the addition of dienogest or its absence, while the addition of HMGB1 promoted decreased expression of these receptors [77].

Another research group extracted lymphocytes from normal endometrium and ovarian endometriomas to examine the expression of IL-4, IL-13, IFN-γ, TGF-β1, and IL-33 receptor by regulatory T cells from these tissues. Treg cells co-cultured with endometriotic stromal cells and anti-IL-33 antibodies produced significant amounts of IL-4, IL-13, TGF-β1, and ST2 (IL-33 receptor). Colocalization of IL-33 and FOXP-3 was observed. In this study, IL-33 from endometriotic stromal cells caused Treg differentiation into Th2-like cells with a concomitant increase in TGF-β1 production, which promoted fibrogenesis [78].

Innate lymphoid cells (ILCs) are a recently discovered family of cells of the innate immune system that are responsible for the rapid immune response and, through the secretion of cytokines, control both the innate and adaptive immune responses. ILCs can be divided into populations according to the main cytokines secreted: IFNγ by ILC1; IL-4, interleukin-5 (IL-5), IL-8, IL9, and IL-13 by ILC2; and IL-17 and IL-22 by ILC3. One study showed that the proportion of ILCs was higher in patients with ovarian endometriomas compared to endometrial samples from patients with endometriosis, and that ILC2 was increased only in ovarian cysts and decreased in endometriosis. Additionally, ovarian cyst tissue contained higher levels of IL-1β and IL-23. No statistically significant differences were described in the peripheral blood or peritoneal fluid, so these observations indicated local inflammation in patients with ovarian cysts in the course of endometriosis [79].

**Figure 3:**
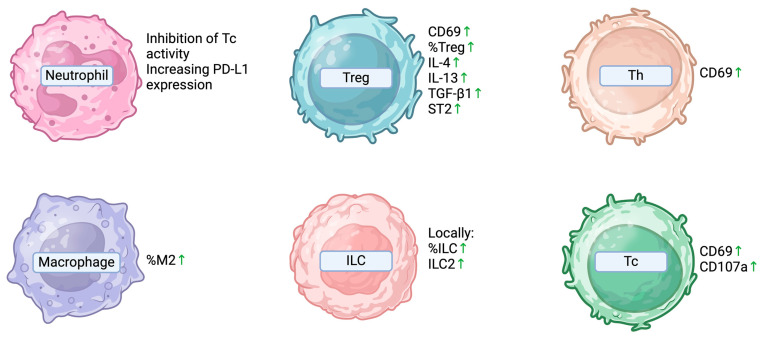
Immune cell abnormalities associated with endometriomas. CD69—cluster of differentiation 69; CD107a—cluster of differentiation 107a/Lysosomal-associated membrane protein 1; IL-4—interleukin 4; IL-13—interleukin 13; ILC—innate lymphoid cells; ILC2—innate lymphoid type-2 cells; M2—M2 polarized macrophages; PD-L1—programmed death 1 ligand; ST2—interleukin 1 receptor-like 1; Tc—cytotoxic T-cells; Treg—regulatory T-cells; TGF-β1—transforming growth factor β1.

Lastly, some evidence suggests endometrioma-associated neutrophils establish an immunosuppressive microenvironment by inhibiting the activity of CD8+ T-cells and increasing PD-L1 expression [80]. Furthermore, in vitro evaluation of neutrophils conducted by Takamura et al. indicated that IL-17A produced by neutrophils stimulated the secretion of Gro-α by the primary culture of endometrial stromal cells [32] (Figure 3).

## 5. NF-κB Pathway

Nuclear factor kappa-B (NF-κB) plays a crucial role in the immune system, primarily by influencing the survival, differentiation, and proliferation of immune cells. Disturbances of its function are the basis of many diseases. Therefore, the postulated involvement of disturbances in its pathway in endometriosis is not surprising. In the case of endometrial cysts in the ovary in advanced endometriosis, researchers have demonstrated a significant increase in the immunoreactivity of the NF-κB p65 subunit compared to control groups. This hyperactivity of NF-κB p65 has also been observed in the eutopic endometrium. Furthermore, following in vitro stimulation of endometrial cells with TNF-α and IL-1β, increases in p65 subunit expression and DNA binding were observed [81].

In another study, endometrial tissue was collected from ovarian cysts in patients who did not receive preoperative hormonal treatment and then used for experiments. IL-6 levels were measured in stromal cell supernatants, showing that this effect was inhibited by the addition of NF-κB and MEK inhibitors and the addition of TNF-α significantly amplified IL-6 secretion. Therefore, the involvement of the NF-κB and MAPK pathways in TNF-α-inducible IL-6 upregulation has been proven [48].

One of the mechanisms that regulate the inflammatory response through NF-κB signaling is endoplasmic reticulum (ER) stress. ER stress can activate the NF-κB pathway in endometrial cells and this signaling induces the production of pro-inflammatory cytokines. In the ovarian endometriotic cyst stromal cell model, upregulation of ER stress by tunicamycin significantly reduced IL-6 and COX2 production by inhibiting NF-κB activity. The authors showed that progesterone treatment did not influence ER stress-induced NF-κB activity or pro-inflammatory cytokine expression in vitro. Taken together, these results suggested that NF-κB activity in endometriotic stromal cells is not inhibited due to an abnormal ER stress response to progesterone, resulting in an increase in proinflammatory cytokine production [49].

## 6. Other Factors

### 6.1. Lipopolysaccharide

Authors demonstrated that lipopolysaccharide (LPS), a bacterial endotoxin, can induce a cytokine cascade in endometriosis. In one study, it was discovered that LPS stimulated TNFα and IL-8 expression with subsequent NF-κB activation, which promoted endometriotic stromal cell (derived from chocolate cysts) proliferation [82].

### 6.2. ICAM-1

Another molecule of interest is ICAM-1, which functions as a regulator of leukocyte recruitment to the site of inflammation. Research by one group showed that, in women with endometriosis, healthy-looking peritoneal tissues but also ovarian endometrial cysts showed high expression of ICAM-1 mRNA. Visually healthy peritoneal cells in patients with endometriosis had high expression of the soluble form of the protein (sICAM-1) without cytokine stimulation, while the eutopic endometrium showed lower expression of sICAM-1. In cultured stromal cells of the eutopic endometrium, ovarian endometriomas, and peritoneal endometriotic spots, ICAM-1 expression increased after INF-γ stimulation. High ICAM-1 expression in healthy peritoneal tissue might play a significant role in early peritoneal endometriosis. The authors postulate that this differential protein expression and its variability may be one of the mechanisms responsible for escape from immunosurveillance and the ability to form endometrial implants [83].

### 6.3. HMGB1

To investigate the role of high-mobility group box-1 protein (HMGB1), which is a danger signal released by damaged tissues and induces inflammatory processes in endometrial cysts, scientists examined the expression of HMGB1 in various cells from women with ovarian chocolate cysts. Tissue samples were collected from patients during surgery. This study showed that dendritic cells (DCs), macrophages, and non-immune cells from endometriomas (such as endometriotic epithelial cells, stromal cells, fibrotic cells, and vascular cells) showed significantly higher cytoplasmic levels of HMGB1 than in the control group. Moreover, the authors showed that the level of this protein could be (at least partially) correlated with tumor size and r-ASRM value [76].

### 6.4. Glycodelin

The evaluation of endometrial flushing fluid from patients diagnosed with endometriomas revealed an increased level of glycodelin, a glycoprotein belonging to the lipocalin family [84,85]. Glycodelin plays a multifaceted role, not only modulating several processes crucial for reproductive health but also acting as a potent immunomodulator [85,86]. Particularly, glycodelin A, its main isoform in the uterine cavity, exhibits immunosuppressive effects on T cells, B cells, and NK cell activity [87]. The exact role of elevated levels of glycodelin in the pathogenesis of endometriomas remains to be fully understood. Nevertheless, the immunosuppressive role of isoform A might be associated with the inflammation present in the reproductive organs of women diagnosed with endometriomas, potentially aiding in the ectopic endometrium’s evasion from immunosurveillance. Moreover, increased levels of glycodelin themselves might negatively impact the rate of successful fertilization and/or pregnancies, which is further decreased by the inflammatory milieu accompanying endometriomas [88].

## 7. Conclusions

Endometriomas are the most prevalent manifestation of endometriosis, a widespread condition that affects a significant percentage of the female population globally. Despite ongoing research, the precise pathogenesis remains not fully understood. In this article, we explore the immune dysregulation accompanying this condition. Notably, current evidence suggests a myriad of factors linked to the inflammatory milieu associated with endometriomas. While these factors could theoretically be categorized into two major groups—local and systemic—it would be artificial to divide them as such. The components of the immune system form a complex web of interplaying factors, and although the endometrioma itself is contained within the walls of the cyst, the immunological dysfunctions appears to be significantly more intricate than merely local inflammation. Moreover, it seems that there is a bilateral connection between the inflammatory environment and endometriomas. It appears that not only do local and systemic inflammation play roles in the etiopathogenesis of endometriomas, but the endometrioma itself is probably capable of inducing systemic and local inflammation.

## Figures and Tables

**Table 1:** Main theories on pathogenesis of endometriosis.

Theory	Mechanism
Retrograde menstruation theory	Retrograde menstruation facilitates the migration of endometrial gland and stromal cells into the peritoneal cavity.
Coelomic metaplasia theory	Metaplasia of the peritoneal serosa into endometrial-like cells and structures can occur due to their common origin as derivatives of the coelomic wall.
Embryogenetic theory	Remnants of embryonic cells present within other structures may develop into endometrial-like cells and structures under the influence of estrogen exposure.
Lymphatic/hematogenous spread theory	Endometrial cells have the potential to travel through lymphatic vessels and/or blood circulation, allowing for their dissemination throughout the body.
Stem cell theory	Multipotent stem/progenitor cells retain the ability to transform into endometrial cells regardless of their location within the body.
Inflammatory/immune dysregulation theory	Endometriosis can develop due to both local and systemic inflammation resulting from complex dysregulation of the immune system.
